# Resection of an intrapulmonary solitary fibrous tumor in the center of the middle lobe: a case report

**DOI:** 10.1093/jscr/rjac466

**Published:** 2022-10-12

**Authors:** Yoshimitsu Hirai, Yoshifumi Iwahashi, Miwako Kogure, Aya Shima, Rie Nakamura, Issei Hirai

**Affiliations:** Department of Thoracic and Cardiovascular Surgery, Wakayama Medical University, Wakayama, Japan; Division of Pathology, Naga Municipal Hospitral, Wakayama, Japan; Department of Respiratory Medicine, Naga Municipal Hospitral, Wakayama, Japan; Department of Breast and General Thoracic Surgery, Naga Municipal Hospital, Wakayama, Japan; Department of Breast and General Thoracic Surgery, Naga Municipal Hospital, Wakayama, Japan; Department of Breast and General Thoracic Surgery, Naga Municipal Hospital, Wakayama, Japan

## Abstract

A 75-year-old female patient was referred to our hospital due to an abnormal shadow detected by chest X-ray. Computed tomography scans revealed a well-circumscribed nodule measuring 28 mm between B4 and B5 in the right middle lobe. Because the tumor was in the center of right middle lobe, a middle lobe resection was performed. The tumor was located within the lung and there were no obvious pleural surface changes. Postoperative histological findings showed 34-mm firm and round tumor, and well circumscribed without involving the visceral pleura. The pathologic examination revealed proliferating spindle-shaped cells with a random fascicular arrangement with continuity to the pulmonary interstitium. Not much cellular atypia was observed. Immunohistochemical staining indicated that the tumor was positive for STAT6, CD34. The final diagnosis was an intrapulmonary benign solitary fibrous tumor (SFT). Even benign intrapulmonary SFTs that have been completely resected may later become malignant and recur, and careful follow-up is necessary.

## INTRODUCTION

Solitary fibrous tumor (SFT) is mostly pleural in origin, most of which develop outside the lung or in the chest wall, and intrapulmonary SFT is relatively rare [1]. Preoperative diagnosis of intrapulmonary SFT is difficult due to their rarity and nonspecific imaging. We report a rare case of intrapulmonary SFT diagnosed after thoracoscopic resection.

## CASE REPORT

**Figure 1:**
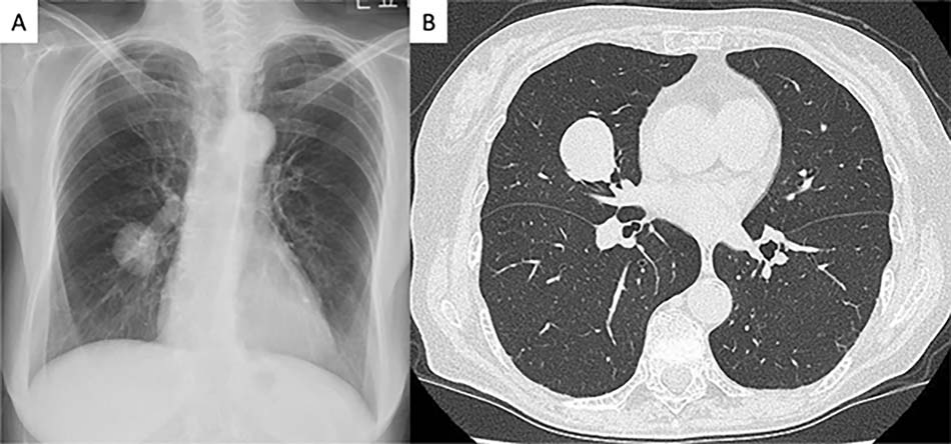
Radiologic findings (**A**) Chest radiograph showed a round mass in the right middle lung field. (**B**) Computed tomography image showing a 28-mm nodule in the middle lobe of the right lung.

A 75-year-old female patient without any symptoms was referred to our hospital due to an abnormal shadow detected by chest X-ray. Laboratory test results, including tumor markers, were all normal. It had at first been detected 3 years earlier and since the tumor was well demarcated, it was suspected to be a benign lesion such as hamartoma, and the patient underwent a conservative follow-up (Fig. 1A). The tumor size gradually increased on during follow-up and subsequently reached from 20 to 28 mm 3 years after the initial imaging (Fig. 1B). Computed tomography scans revealed a well-circumscribed nodule measuring 28 mm between S4 and S5 in the right middle lobe. Other tests, including serum tumor markers, were within normal limits. We recommended preoperative 18F-fluoro-deoxy-glucose positron emission tomography and bronchoscopy, but the patient refused to undergo preoperative examinations.

Because the tumor was in the center of right middle lobe, enucleation or partial resection was difficult to perform. Middle lobe resection was performed for diagnosis and treatment. The surgery was performed using a complete thoracoscopic approach with four ports (7, 7, 15 and 30 mm). The tumor was located within the lung and there were no obvious pleural surface changes. The total operation time was 150 min and the bleeding amount was 15 ml. She had no postoperative complications and was discharged 7 days after surgery.

**Figure 2:**
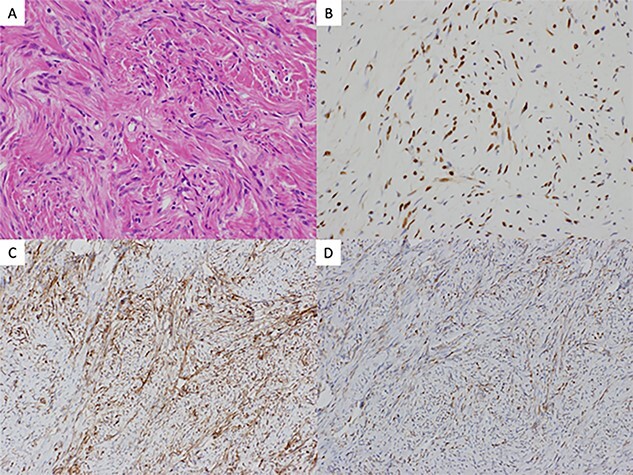
(**A**) histology hematoxylin and eosin staining of the resected tumor revealed proliferating spindle-shaped cells with a random fascicular arrangement with continuity to the pulmonary interstitium (×40). Immunohistochemical findings revealed the tumor cells to be positive for (**B**) STAT6 (×200), (**C**) CD34 (×200), and (**D**) bcl-2 (×200)

Postoperative histological findings showed 34-mm firm and round tumor, and well circumscribed without involving the visceral pleura. The pathologic examination revealed proliferating spindle-shaped cells with a random fascicular arrangement with continuity to the pulmonary interstitium (Fig. 2A). Not much cellular atypia was observed. Immunohistochemical staining indicated that the tumor was positive for STAT6 (Fig. 2B), CD34 (Fig. 2C), bcl-2 (Fig. 2D) but negative for cytokeratins, EMA, CD99, S-100. The WHO classification for SFT grade uses the presence of dense cellular regions and multiple fissions (>4 mitoses/2 mm^2^) as diagnostic criteria [2]. In this case, there was no visible necrosis and mitoses were infrequent. The final diagnosis was an intrapulmonary benign SFT. Three months after the surgery, the patient is under outpatient follow-up with no recurrence.

## DISCUSSION

SFT was described as a pleural lesion by Klemperer *et al*. [3] in 1931 but is a tumor with an incidence of 2.8 per 100 000 persons. Two-third of SFTs arise from the visceral pleura and one-third from the mural pleura, and locations other than the pleura such as the peritoneum, pericardium, lung parenchyma, mediastinum, and pelvis have also been reported [4]. As for the origin of intraplmonary SFT, Aufiero *et al*. [5] reported two hypotheses: (i) the interlobar pleura and the lung parenchyma just below the pleura traffic, and tumors arise from stromal cells in the lung and (ii) tumors arise from fibroblasts in the lung parenchyma itself. When intrapulmonary SFTs are in contact with the visceral pleura, it suggests that the tumor is of visceral pleural origin, which is no different from the usual origin of SFTs. In the present case, the tumor was located in the center of the middle lobe and most likely arose from an origin not related to the pleura at all. Most of the reported cases also involved tumors that were in contact with the visceral pleura, including the interlobar pleura, and intrapulmonary SFTs that were not in contact with the pleura were considered to be relatively rare.

Immunohistochemical testing is useful in differentiating SFT from other neoplasms. CD34 immunoreactivity has traditionally been used for differential diagnosis but is not specific. Recently, nuclear staining for STAT6 has been established as a more sensitive and specific marker and is widely used [6]. In the present case, it was also extensively positive.

The standard treatment for SFT is complete surgical resection. Although lobectomy was selected in this case due to the location of the tumor, in reality, a reduction surgery such as a segmentectomy or partial resection is often selected because a low-grade tumor is suspected on intraoperative rapid pathological diagnosis. Most SFTs have a clinically benign course but may metastasize or recur postoperatively [7, 8]. Rao *et al*. [9] reported 24 cases of intrapulmonary SFTs, all of which were resected. Histopathologically, based on cellular atypia and nuclear fission image, low grade (<5/10 high power field (HPF)) was found in 21 cases, intermediate grade (5–10/10 HPF) in 1 case and high grade (>10/10 HPF) in 2 cases. Of the 18 cases that were traceable, 3 died, 1 was high grade and 2 low grade recurred with malignant transformation and died. All 14 recurrence-free cases were low grade. Inoue *et al*. [10] also reported a partial resection of a benign intrapulmonary SFT with 2/10 HPF that recurred 2 years later and was re-resected. Ozeki *et al*. [11] reported a case of intrapulmonary SFT that recurred with brain metastasis 7 months after resection. Therefore, it is necessary to keep in mind that intrapulmonary SFT is a differential diagnosis for well-defined tumors in the lung and even benign intrapulmonary SFTs that have been completely resected may later become malignant and recur, and careful follow-up is necessary.
